# Does spatial perspective in virtual reality affect imitation accuracy in stroke patients?

**DOI:** 10.3389/frvir.2022.934642

**Published:** 2022-09-07

**Authors:** Erica M. Barhorst-Cates, Mitchell W. Isaacs, Laurel J. Buxbaum, Aaron L. Wong

**Affiliations:** Moss Rehabilitation Research Institute, Philadelphia, PA, United States

**Keywords:** imitation, virtual reality, spatial perspective, stroke, apraxia

## Abstract

Imitation is an important daily activity involved in social interactions, motor learning, and is commonly used for rehabilitation after stroke. Moreover, deficits in imitation of novel movements commonly occur after left hemisphere stroke (LCVA) in the syndrome of limb apraxia. In the current study, we used a novel virtual reality (VR) imitation paradigm to assess two factors that have remained underexplored in novel movement imitation: the imitation of complex, dynamic full-arm movements, and the effect of spatial perspective. VR holds promise as a tool for a number of clinical assessments and treatments, but has very rarely been studied in the context of imitation or diagnosis of apraxia. Thirty participants (18 with LCVA and 12 age- and education-matched controls) wore a VR headset and observed and imitated an instructor avatar demonstrating arm movements. Three spatial perspectives were examined within-subjects: first-person, third-person mirror, and third-person anatomical. Movements of the ipsilesional (left) arm were recorded and qualitatively coded for accuracy compared to the instructor avatar. Participants also completed embodiment questionnaires, a measure of limb apraxia (imitation of video-recorded meaningless movements), and three computerized background tasks that were hypothesized to evoke some of the same processing requirements of each of the three perspective conditions: a block-matching task, a block-mirroring task, and a mental rotation task. Imitation accuracy was highest in the first-person perspective, consistent with predictions, but did not differ between third-person mirror and anatomical. Surprisingly, patients and controls performed similarly on the imitation task for all spatial perspectives, with overall modest accuracy in both groups, and both patients and controls felt a moderate level of embodiment of their own avatar. Higher imitation accuracy related to quicker block-matching reaction times and higher mental rotation accuracy, regardless of perspective, but was unrelated to imitation of video-recorded meaningless movements. In sum, virtual reality provides advantages in terms of experimental manipulation and control but may present challenges in detecting clinical imitation deficits (limb apraxia).

## 1 Introduction

Imitation is an important daily activity involved in social interactions, motor learning, and is commonly used for rehabilitation after stroke (Franceschini et al., 2010; Small et al., 2012; Hatem et al., 2016). However, deficits in the imitation of novel movements and postures are a hallmark of the clinical disorder of limb apraxia, present in approximately 30%–50% of individuals with cerebrovascular accident in the left hemisphere (LCVA; De Renzi et al., 1980; Donkervoort et al., 2000). Notably, apraxia can be observed in both the contralesional (affected) and ipsilesional limbs, but is usually assessed in the ipsilesional limb since motor deficits can limit the use and precision of the contralesional limb (Buxbaum and Randerath, 2018). Both clinical and research assessments of apraxia often involve imitating static postures or dynamic movements presented on video by copying an instructor who is seated across from the patient as if looking in a mirror (i.e., the instructor demonstrates with their right arm and the patient imitates with their left arm). In the current study we used a novel virtual reality (VR) imitation paradigm to assess two factors that have remained underexplored in novel movement imitation: the imitation of complex, dynamic full-arm movements, and the effect of spatial perspective. Using VR provided two main advantages: it allowed us to 1) assess a new potential tool for identifying apraxic impairments and 2) directly compare a condition that would not be possible to test in the real world (i.e., movements cued from a true first-person perspective) to those of mirroring and anatomical imitation.

Imitation is a common component of rehabilitation for many individuals following a cerebrovascular accident (CVA; commonly referred to as “stroke”). For example, learning new strategies for performing everyday actions, such as making a cup of coffee or buttoning a shirt, may require an individual with CVA to first observe a therapist or caregiver demonstrating a new technique and then attempting to imitate it. For the sake of simplicity, we will hereafter refer to the person demonstrating the actions as the “instructor” and the person observing and imitating the movements as the “observer.” There are at least three ways that an instructor could demonstrate an action to an observer. In general, when observing an instructor demonstrating a movement, observers tend to perform an egocentric transformation to map the movement onto their own body (Kessler and Thomson, 2010; Creem-Regehr et al., 2013). Some perspectives require more complex transformations than others, which can affect the difficulty of the imitation task. In the first-person perspective, the observer views an instructor demonstrating an action from their own perspective, or from a perspective in line with what they are accustomed to, and imitates the action from that same perspective. For example, the observer could view photos or videos of an instructor’s hands performing an action from their own perspective, view the instructor from behind, or be positioned immediately next to the instructor. In each case, this perspective requires few to no spatial transformations in order for the observer to map the movements onto their own body. This advantage of aligned perspectives, termed the spatial compatibility effect, has been observed in several studies (Jackson et al., 2006; Watanabe and Higuchi, 2016) and results in quicker and more accurate imitation.

In the mirroring perspective, the observer views the instructor performing an action and then imitates with the opposite limb/movement directions, as if they were looking in a mirror. This requires a mirroring spatial transformation to map between instructor and observer. Mirrored imitation is a commonly used method in rehabilitation, which may be because it is thought to be natural and automatic (Schofield, 1976; Bekkering et al., 2000; Franz et al., 2007). Indeed, apraxia-related deficits are often measured using tasks such as viewing and imitating pictures or videos (e.g., Goldenberg 1995; Buxbaum et al., 2014) from a mirrored perspective. Mirroring as an instructional movement tool is also used in several fields, including dance-movement therapy (for a review see Fitzpatrick 2018), likely because it is clearly more practical than trying to instruct using a first-person perspective. Whether it provides an equally good cue to the observer, however, remains up for debate. Several studies have directly compared mirroring and first-person perspectives (Jackson et al., 2006; Watanabe et al., 2013; Watanabe and Higuchi, 2016), finding advantages for the first-person perspective due to decreased spatial transformation requirements. Nevertheless, both first-person and mirroring imitation are considered spatially compatible cuing perspectives (i.e., the movement to be imitated is still on the same side of space in both cases; (Chiavarino et al., 2007; Watanabe et al., 2013; Nishizawa et al., 2015).

Finally, in the anatomical perspective, the observer views the instructor performing an action, and then imitates it using the same limbs/movement directions (e.g., both the instructor and the observer produce a movement to their right using their right arm). This may require a complex mental transformation for the observer to map the instructor’s movements onto the observer’s frame of reference (e.g., imagining the instructor rotated by 180°), a process known as spatial perspective taking (Hegarty and Waller, 2004). Larger discrepancies between perspectives require both effortful mental transformation and management of interference stemming from the disparity between instructor and observer movement directions (Brockmole and Wang, 2003; Kessler and Thomson, 2010). One study showed that imitating another’s spatially incongruent perspective is slower and more error-prone than imitation following observation from the observer’s first-person perspective because there is a less direct mapping between the demonstrated movement and the observer’s own body (Krause and Kobow, 2013). Similarly, anatomical imitation has also been shown to increase imitation errors compared to mirroring due to the lack of spatial compatibility (Chiavarino et al., 2007; Nishizawa et al., 2015).

Though the evidence suggests that imitation from a first-person perspective is advantageous over any third-person perspective, in traditional settings there are times when it is not feasible to use the first-person perspective (such as when conducting a rehab session over video or taking an online dance class). Sometimes the instructor also needs to be able to view the observer to adapt and correct their movements, which is not possible when the first-person perspective is provided by observing the instructor from behind. Moreover, if the observer is positioned behind the instructor, parts of the movement may not be clearly viewable. Here we examine two alternatives to address these problems with the first-person perspective. On one hand, a third-person perspective may be used instead, as noted above. For this solution, some evidence suggests that mirroring may be preferable to anatomical imitation due to the greater spatial compatibility inherent in the former (Chiavarino et al., 2007; Nishizawa et al., 2015). Indeed, individuals with CVA demonstrate advantages for the mirroring perspective over anatomical, and those with frontal lobe lesions may be particularly impaired on anatomical imitation (Chiavarino et al., 2007). As such, individuals with CVA should theoretically benefit most from imitation situations that are the most spatially compatible (requiring the fewest spatial transformations). However, to our knowledge the two third-person perspectives and the first-person perspective have not been directly compared in terms of their effectiveness in a patient sample (but see Nishizawa et al., 2015 for a study with neurotypicals).

A second possible solution to the limitations of the first-person perspective lies in the provision of a more realistic first-person perspective through the use of immersive virtual reality (i.e., the participant is wearing a headset that projects them into the virtual environment). Virtual reality (VR) has received much attention for its potential in rehabilitation (for reviews see Schultheis and Rizzo 2001; Rose et al., 2018; Bevilacqua et al., 2019), but there are relatively few studies using VR in stroke populations, and many of those have used desktop-based VR applications rather than immersive VR (e.g., (Buxbaum et al., 2012; Alarcón-Aldana et al., 2020). Immersive VR offers many advantages over other testing and assessment tools. To optimally assess imitation and other clinically-relevant behaviors in VR, participants should perceive the environment and avatar as naturally as possible. Generally, VR has the potential to induce illusory ownership of virtual arms (Slater et al., 2008) and virtual bodies (Slater et al., 2009). Taking a first-person perspective induces a greater sense of body ownership of a virtual avatar (Petkova et al., 2011), which contributes to avatar embodiment, or the sense that one’s avatar has replaced one’s real body in the virtual environment (Gonzalez-Franco and Peck, 2018; Peck and Gonzalez-Franco, 2021). Immersive VR also allows a unique opportunity for testing imitation from the first-person perspective (i.e., viewing one’s own avatar demonstrating the movement). Previous research examining imitation with the first-person perspective have either used static photos or video-based paradigms where the participant viewed another individual from behind (Kessler and Thomson, 2010), or movements were limited to those that could be discerned easily in 2D (e.g., viewing only the hands; (Jackson et al., 2006). Furthermore, prior studies have used simple finger-tapping movements (Watanabe et al., 2013; Watanabe and Higuchi, 2016) or position-matching tasks (Krause and Kobow, 2013) that may not reflect complex, real-world movement. In contrast, immersive VR allows a unique situation that can test a true first-person perspective more explicitly and in 3D, by having the observer’s own avatar demonstrate the movement. When observing one’s own avatar moving in immersive VR, the participant can look down at their body and turn their head to view full arm movements that would not be possible to present in a video (e.g., movements that reach far to the upper right and then to the lower left). This affords the ability to examine imitation of full-arm motion in a large region of space. Another advantage of using immersive VR to test the first-person perspective is the ability to directly match the observation and performance environments (e.g., the participant can both view and perform movements in the virtual environment, rather than switching between observing on video/photos then repeating the movement in the real-world). Thus VR offers several advantages for experimental manipulation and has potential as a diagnostic tool for assessing apraxia-related imitation deficits.

The current study therefore aimed to compare these alternatives directly in a patient sample. Here we designed a novel task with challenging, realistic full arm movements with the goal of identifying which imitation perspectives may be optimal for measuring imitation ability in individuals with left CVA (LCVA) compared to a group of age- and education-matched neurotypical controls. Broadly, we predicted that imitation accuracy across all individuals would be highest in the first-person condition, followed by the mirroring condition, followed by the anatomical condition, consistent with the relative increases in spatial transformation requirements. We also expected that our imitation paradigm in VR would reveal imitation deficits at the clinical level, reflecting the degree of limb apraxia in our population. As such, we predicted that controls would perform more accurately than individuals with LCVA—especially those with limb apraxia—in all conditions, and that imitation accuracy in our VR task would be correlated to a more traditional measure of limb apraxia. This would suggest that our VR paradigm was sufficiently sensitive to detect apraxic impairments in a patient sample. We also included several additional measures to attempt to characterize the processes involved in imitation in VR. Participants completed a brief embodiment questionnaire after each condition as well as three computerized background tasks that were hypothesized to evoke some of the same visuospatial processing requirements of each of the three perspective conditions: a standard mental rotation task, a novel block-mirroring task, and a novel block-matching task. Mental rotation is a well-researched visuospatial task that assesses spatial transformation abilities, requiring an individual to imagine the rotation of objects to determine whether they are the same or different (e.g., Shepard and Metzler 1971; Zacks 2008). As such, we expected mental rotation to be an analogous measure to the anatomical condition in our study. We did not know of any similar tasks to assess the visuospatial processes in mirroring and first-person imitation, so we created analogous block-mirroring and block-matching tasks that required participants to select mirror-images or exact matches compared to a target object, where objects consisted of arrangements of blocks similar to those presented in conventional mental rotation tasks. Together, these efforts provided insights for applying virtual reality to assess limb apraxia following a left-hemisphere stroke.

## 2 Method

### 2.1 Participants

We determined our goal sample size based on prior studies that have examined imitation perspectives which included samples ranging from 15 to 34 neurotypical individuals (Jackson et al., 2006; Kessler and Thomson, 2010; Krause and Kobow, 2013; Nishizawa et al., 2015; Watanabe and Higuchi, 2016) and one study with patients that included 16 patients and 7 controls (Chiavarino et al., 2007). Moreover, we followed the standard in patient research to include a larger sample of patients relative to controls, given the expectation of greater inter-subject variability in the patient group (Chiavarino et al., 2007; Wong et al., 2019; Isaacs et al., 2021). Thus, in line with these prior studies, 30 participants completed the study: 18 individuals with LCVA (8 male) and 12 controls (4 male). The average age of the individuals with LCVA (*M* = 61.8, *SD* = 11.4) and controls (*M* = 62.7, *SD* = 11.2) did not significantly differ according to a *t*-test [*t* (76.82) = 1.08, *p* = 0.286]. All participants were recruited through the Moss Rehabilitation Research Registry and were compensated $15 per hour for their time. All participants provided written informed consent with procedures approved by the Institutional Review Board of Einstein Healthcare Network.

### 2.2 Materials and procedure

#### 2.2.1 Virtual reality imitation task

Researchers attached kinematic motion trackers to the participant’s left (ipsilesional) thumb, pointer finger, upper hand, forearm, and upper arm using surgical tape and elastic bands. Movements were tracked using a magnetic motion tracking system (trakSTAR, Northern Digital Inc., Waterloo, Ontario, CA,United States). We ensured that each participant retained full range of motion of the arm and felt comfortable moving as they normally would. Each participant also wore a small strap with a 6^th^ tracker centered on their upper back. These trackers were used to record the participants’ movements and provide visual feedback of movement within the headset. Participants were seated throughout the experiment in an armless chair and were told to return their left hand to their left leg at the end of each movement.

Participants completed the VR movement imitation task programmed in Unity while wearing a virtual reality headset (HTC Vive). We selected an avatar that matched the participant’s gender and race. We included 4 avatar options that reflected the demographics of our research registry population: a white woman, a white man, a black woman, and a black man. Avatars were downloaded from Modern People 2 in the Unity Asset Store. In all conditions, identical avatars were used to represent the instructor and the observer. For initial calibration, the experimenter used the Vive controller to adjust the motion of the observer avatar’s arm to match the actual motion of the participant’s arm using a custom system wherein the position of each tracker was manually aligned in the virtual world to the position of the participant’s body in the real world. Using the controller, the experimenter selected each relevant joint in turn (thumb, pointer finger, upper hand, forearm, upper arm, and upper back) and adjusted each joint’s position to match the real-world position of the participant’s arm at rest. The rotation offsets for each joint were saved in the program and maintained throughout the experiment for each participant. We then told participants that they would be viewing movements and repeating them: they would see a virtual instructor demonstrating movements with the right or left arm, and they were to watch the movement, then repeat it back at the same speed using their left arm. Instructor movements lasted approximately 3–5 s and participants began imitating the movement immediately after the instructor finished demonstrating. There were three perspective conditions: first-person, mirroring, and anatomical.

In the *first-person* condition, the instructor avatar was positioned in the same location as the participant, such that it appeared as if the participant’s own avatar’s arm was moving. This required the participant to look down at their own virtual body and observe the virtual arm moving while keeping their real arm still. Then they imitated the observed action. For example, if the instructor avatar performed a V movement with their left arm by first elevating it above the left shoulder, lowering it to midline, then elevating it above the right shoulder, the participant should copy the movement exactly by extending their left arm and elevating it above the left shoulder, lowering it to the midline, then elevating it above the right shoulder. During a pre-experiment instruction period, the experimenter demonstrated this by kneeling directly next to the participant and demonstrating movements with their arm extending from the participant’s shoulder to emphasize that it would be the participant’s avatar demonstrating the movements.

In the *mirroring* condition, the instructor avatar was seated directly across from the participant in a chair. The participants were instructed to observe the movement, then imitate it as if they were looking in a mirror. For example, if the instructor avatar performed a V movement with their right arm by first elevating it above the right shoulder, then lowering it to midline, then elevating it above the left shoulder, the participant was asked to perform the same V movement with their left arm as in the example above (i.e., mirroring the movement). During a pre-experiment instruction period, the experimenter demonstrated a movement with their right arm while kneeling in front of the participant and had the participant practice mirroring it with their left arm.

**FIGURE 1:**
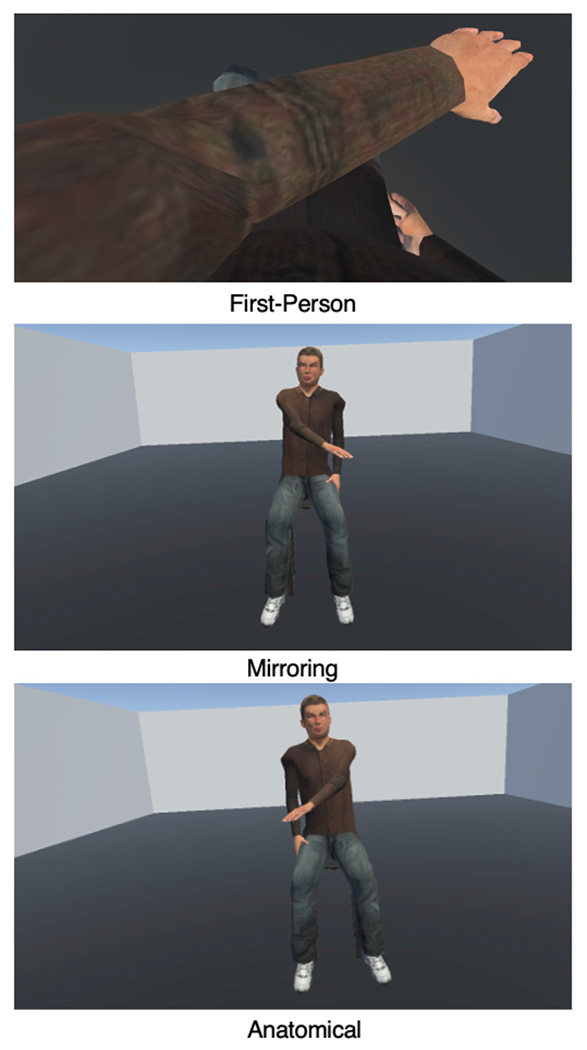
Screenshots of the three conditions from the perspective of the participant. In the first-person condition, participants looked down at their own body and viewed their own avatar demonstrating the movement with the left arm. In the mirroring condition, participants viewed an avatar seated across from them demonstrating a movement with the right arm, as if looking in a mirror. Finally, in the anatomical condition, participants viewed an avatar seated across from them demonstrating a movement with the left arm. The participant was then required to imitate the observed action using their own left arm; hence in all three of these examples, the participant’s movement would be the same.

Finally, in the *anatomical* condition, participants were instructed to observe the movement of the instructor (who was again seated across from the participant), then imitate it with the same arm as the instructor avatar. In the case of the V movement, the instructor would demonstrate the movement with their left arm, and the participant would also perform the movement with their left arm identically as in the two examples above. Note, however, that from the observer’s perspective, anatomical imitation of the action required a 180° rotation of perspective relative to the instructor. During a pre-experiment instruction period, the experimenter demonstrated a movement with their left arm, and asked the participant to practice copying the same movement with their left arm. See Figure 1 for an overview of these three conditions.

Prior to each block, participants practiced 2 movements in the upcoming condition while wearing the headset until they performed the movement accurately. Participants then completed 12 different movement trials in each condition. Movements ranged in difficulty by consisting of 1 or 2 movement segments. All participants completed all three perspective conditions, each involving the same 12 movements presented in a different random order. Each movement was qualitatively coded for accuracy (see below) and analyzed separately. Test conditions were blocked and the order of blocks was counterbalanced between participants.

#### 2.2.2 Embodiment questionnaire

Following completion of each condition, participants responded aloud to a brief embodiment questionnaire to assess how realistic they perceived the movements to be for their own and the instructor avatars. We created the questionnaire for this study based on previous embodiment questionnaires (Gonzalez-Franco and Peck, 2018; Peck and Gonzalez-Franco, 2021), with modifications to reduce linguistic complexity. We aimed to assess participants’ perception of own-avatar embodiment, own-avatar sense of control, and other-avatar embodiment. We asked participants three questions after each condition, while they were still wearing the headset: 1) Do you feel like the body you see when you look down is your body? 2) When you were performing the movement, did you feel like you could control the virtual body as if it were your own body? 3) When you were watching the movement, did you feel as if the virtual instructor was another person? Participants responded to each question by saying aloud “yes,” “no,” or “sort of.” These responses were then coded numerically on a scale from 0 to 2. We included these simplified response choices, rather than standard 5–7 item Likert scales, to minimize the working memory demands on the participant during their response (especially given they were still wearing the headset and could not look at a written version of the scale). We also included an open-ended question at the end of the embodiment questionnaire where we asked participants if there was anything else they wanted us to know about their virtual body or the virtual instructor. We included this to glean insight into the participants’ experience in VR.

#### 2.2.3 Background tasks

At the end of the VR portion of the study, we removed the headset and transitioned the participant to a desk with a desktop computer. They then completed three background tasks in a random order. We provide a comparative analysis between groups on these measures in the Supplementary Materials.

#### 2.2.4 Mental rotation task

Participants completed the standard mental rotation task in the Psychology Experiment Building Language (PEBL) (Mueller and Piper, 2014). The mental rotation task was included as a background task meant to assess cognitive processes related to the anatomical condition (which we predicted would require a complex spatial transformation/rotation of perspective). Participants viewed two shapes on a computer screen that were rotated with respect to one another and had to decide whether the shapes were the same or different. The objects were rotated either 0°, 45°, 90°, or 135° with respect to each other. The shapes were the same if they could be rotated in the picture plane to match one another. Participants viewed instructions and completed practice trials until they responded with 100% accuracy. Participants were instructed to respond as soon as they had decided whether the objects were the same or different by pressing the ‘S’ or ‘D’ keys on the keyboard, respectively. Accuracy and response time were recorded. The task included 64 test trials, 16 at of the 4 angles of rotation.

#### 2.2.5 Block-matching task

**FIGURE 2:**
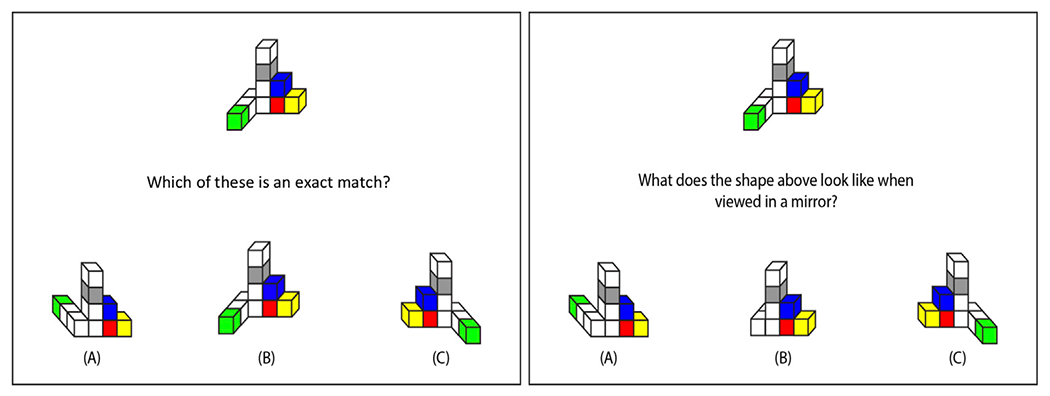
Example trials from block-matching (left panel) and block-mirroring (right panel) tasks. Participants responded on the keyboard to select which of the three bottom figures is an exact match (correct answer is B) or the mirrored version (correct answer is A) of the top figure.

Participants completed a novel computerized block-matching task created for this study, which was designed to assess the visuospatial processes related to the first-person condition. They viewed one configuration of cubes at the top of the screen and three answer choices at the bottom. They were instructed to use the mouse to select which of the three objects at the bottom was an exact match to the target object at the top (Figure 2A). Participants viewed instructions and completed practice trials until it was clear that they understood. Accuracy and response time were recorded. Participants were instructed to respond quickly and accurately. Trials timed out after 20 s if there was no response. The task included 16 test trials.

#### 2.2.6 Block-mirroring task

Participants also completed a novel computerized block-mirroring task created for this study, which was designed to assess the cognitive processes related to the mirroring condition. They viewed one configuration of cubes at the top of the screen and three answer choices at the bottom (see Figure 2). They were instructed to use the mouse to select which of the three objects at the bottom was what the target object would look like in a front-facing mirror (i.e., a mirror placed just behind the object such that the participant could see both the object and its reflection). Participants viewed instructions and completed practice trials until it was clear that they understood. Accuracy and response time were recorded. Participants were instructed to respond quickly and accurately. Trials timed out after 20 s if there was no response. The task included 16 test trials.

#### 2.2.7 Meaningless imitation

In a separate session, individuals with LCVA completed our laboratory’s measure of imitation of meaningless gestures (see Buxbaum et al., 2014 for a description of this paradigm). On each trial participants viewed a video of an instructor performing a meaningless gesture with one arm, then immediately imitated the gesture along with a second viewing of the same video. Gestures were imitated from a mirroring perspective. Participants were filmed and their imitation of each item was coded for accuracy (0 or 1) on arm posture, hand posture, amplitude, and timing components using a well-established coding system used in many prior studies (Buxbaum et al., 2005; Jax et al., 2006; Buxbaum et al., 2007; Kalénine et al., 2013; Garcea et al., 2020). Each gesture received a sum score ranging from 0 to 4 from which we calculated an overall average imitation accuracy. We included the average overall imitation accuracy scores from this task to assess whether the VR task was sensitive to limb apraxia.

## 3 Data analysis

### 3.1 Qualitative coding

Imitation accuracy can be measured and defined in many different ways based on qualitative or quantitative methods, and there is no single solution for performance assessment [for a review, see (Liao et al., 2020)]. Given the variation in anatomies and kinematics between subjects that may preclude accurately quantifying imitation accuracy based on kinematics alone, we took an approach similar to the coding scheme used by Buxbaum et al. (2005). in many studies of imitation deficits in apraxia (Jax et al., 2006; Buxbaum et al., 2007; Kalénine et al., 2013; Garcea et al., 2020). This also allowed us to make more direct comparisons between our data and that of our conventional meaningless imitation task, for which the data were similarly coded.

On the experimental tasks, imitation accuracy was coded manually by two researchers by observing the playback of the kinematic data using custom Matlab scripts. Movements were broken down into component parts: static postures and the dynamic trajectories in between. Each static position or trajectory received a point if it was correct. An accuracy score for each movement was then calculated as the sum of points earned divided by the total number of possible points for that movement. So as to reduce interdependency in scoring postures and trajectories (i.e., to not penalize a participant for an unusual trajectory if the starting or ending position was incorrect), each dynamic trajectory received a point if it was generally correct in terms of shape (straight or curved) and direction, regardless of the start and end location. The researchers first reached agreement on the coding scheme and achieved high inter-rater reliability on a subset of participants (Cohen’s kappa = 0.87). Then the participants’ data were divided between the researchers for qualitative coding. Each movement’s score was converted to a percentage by normalizing the number of points earned by the total number of possible points for that movement.

### 3.2 Statistical analyses

We had four main analyses. In our first analysis, we aimed to determine the effects of group and condition on imitation accuracy, and whether there was an interaction between the two. We used mixed effects modeling, a flexible modeling approach that allows imbalances in data and the inclusion of multiple random effects. We tested fixed effects of group, condition, and the group*condition interaction. Group (patient vs. control) and condition (first-person, mirroring, and anatomical) were each converted to factors prior to analyses. We included random effects of participant and movement number in all mixed effects models. Following standard procedures in mixed effects models, we used likelihood ratio tests to compare models with and without the factor of interest (Peugh, 2010). To interpret the significant factors according to the likelihood ratio test, we performed planned contrasts with a Kenward-Roger approximation to estimate degrees of freedom and a Tukey adjustment for multiple comparisons. We also calculated the effect size for each comparison (Cohen’s D).

In our second analysis, we aimed to determine whether differences in imitation performance were paralleled by differences in the tendency to embody the avatar in the different conditions. We ran linear regressions with group, condition, and the group*condition interaction predicting embodiment for each of the three questionnaire items.

In our third analysis, we aimed to assess the effect of limb apraxia in the patient group. With data from the patients only we used mixed effects models to test fixed effects of condition and meaningless imitation. We again included random effects of participant and movement number.

Finally, to further examine the variation in imitation accuracy throughout the task, we considered how performance on the control tasks related to imitation. Due to experimenter error, there were missing data from 4 participants on the mental rotation task and 2 participants on block-mirroring and block-matching. Because our control tasks included both accuracy and reaction time (RT), we ran a separate model for each of these two variables independently. For each task, we ran a separate linear mixed effects model for imitation accuracy as the dependent variable and condition, average background task accuracy (or RT), and the condition*background task accuracy (or RT) interaction as fixed effects, as well as participant and movement number as random effects. For patient and control differences on each background task, see the Supplementary Materials.

All analyses were performed in R. For mixed effects models, we used *lme4* (Bates et al., 2015), and *lmerTest* (Kunzetsova et al., 2017) with *emmeans* (Lenth, 2019) to perform planned contrasts. For regressions we used the package *stats* (R Core Team, 2018). Plots were created with *ggplot2* (Wickham, 2016).

## 4 Results

### 4.1 Imitation analysis

**FIGURE 3:**
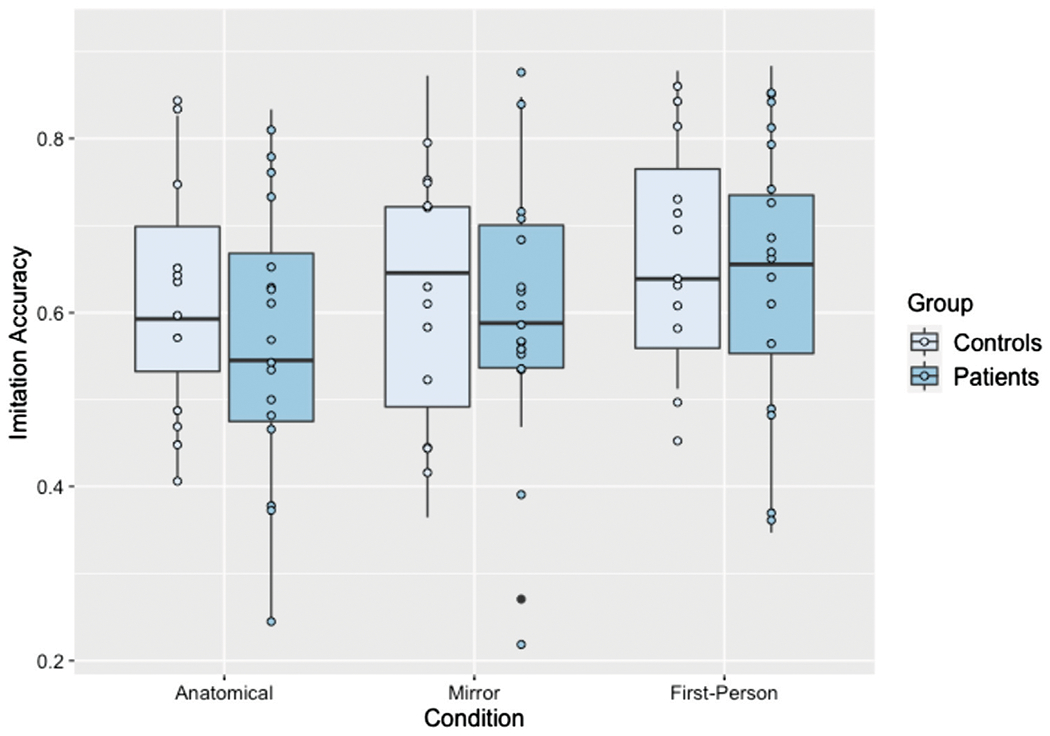
Boxplot of imitation accuracy scores separated by condition and group. Each dot represents an individual participant’s performance in this condition.

Individuals with LCVA and controls performed similarly on the task overall, and imitation accuracy was modest with average accuracy of 0.66 (*SE* = 0.04) in the first-person condition, 0.60 (*SE* = 0.04) in the anatomical condition, and 0.61 (*SE* = 0.04) in the mirroring condition. There was a significant effect of condition [*χ^2^*(2) = 9.86, *p* = 0.007], but no significant effect of group [*χ^2^*(1) = 0.52, *p* = 0.470]. The interaction was also not significant [*χ^2^*(2) = 0.59, *p* = 0.746]. Planned contrasts revealed that accuracy in the first-person condition was significantly higher [*t* (1,011) = −2.91, *p* = 0.010, *d* = 0.22]^1^ than accuracy in the anatomical condition, though accuracy in first-person did not differ significantly [*t* (1,017) = −2.02, *p* = 0.107, *d* = 0.16] from accuracy in the mirroring condition. Accuracy also did not significantly differ between anatomical and mirroring conditions [*t* (1,017) = −0.89, *p* = 0.646, *d* = 0.07]. Thus, imitation accuracy was highest in the first-person perspective, regardless of group. See Figure 3 for a depiction of these results.

### 4.2 Embodiment questionnaire

Both individuals with LCVA and controls reported a moderate level of embodiment (the sum score of the three items) of their own avatar (*M* = 1.37 out of 2, *SD* = 0.87). However, we did not observe any significant effect of group or condition on the degree of reported embodiment (*ps* > 0.3), despite qualitatively reporting greater ownership of the avatar in the first-person condition (see below). See Table 1 for average responses on the embodiment questionnaire in each group and condition.

In general, qualitative interviews revealed that many participants found the task to be hard. One participant commented, it would be “different if it were a real person. If [they] were more experienced with VR, it might be different.” Participants commented on how the avatar “sometimes d[id]n’t look exactly right” and “moved different from [them].” Nevertheless, participants commented that in the first-person condition the instructor “felt like [them]” and in comparison was “so different than other [conditions]; now, [they] feel like this is [their] arm.” In contrast, during the anatomical condition, participants commented on how it was “hard to think of [the] rotation” and that the instructor was “hard to imitate; hard to remember,” and although one participant “liked [the mirroring condition] better,” they commented that it was still “harder than [they] thought.” In sum, participants found the task to be quite difficult, which is reflected in the moderate accuracy scores.

### 4.3 Relationship between imitation accuracy and limb apraxia measure

In the patient data, there was no significant effect of meaningless imitation on VR imitation accuracy (*χ^2^*(2) = 0.70, *p* = 0.402), suggesting that regardless of the spatial perspective, the VR imitation task may not have been as sensitive to detecting limb apraxia in comparison to our standard measure.

### 4.4 Background visuospatial tasks and imitation accuracy

#### 4.4.1 Mental rotation

**FIGURE 4:**
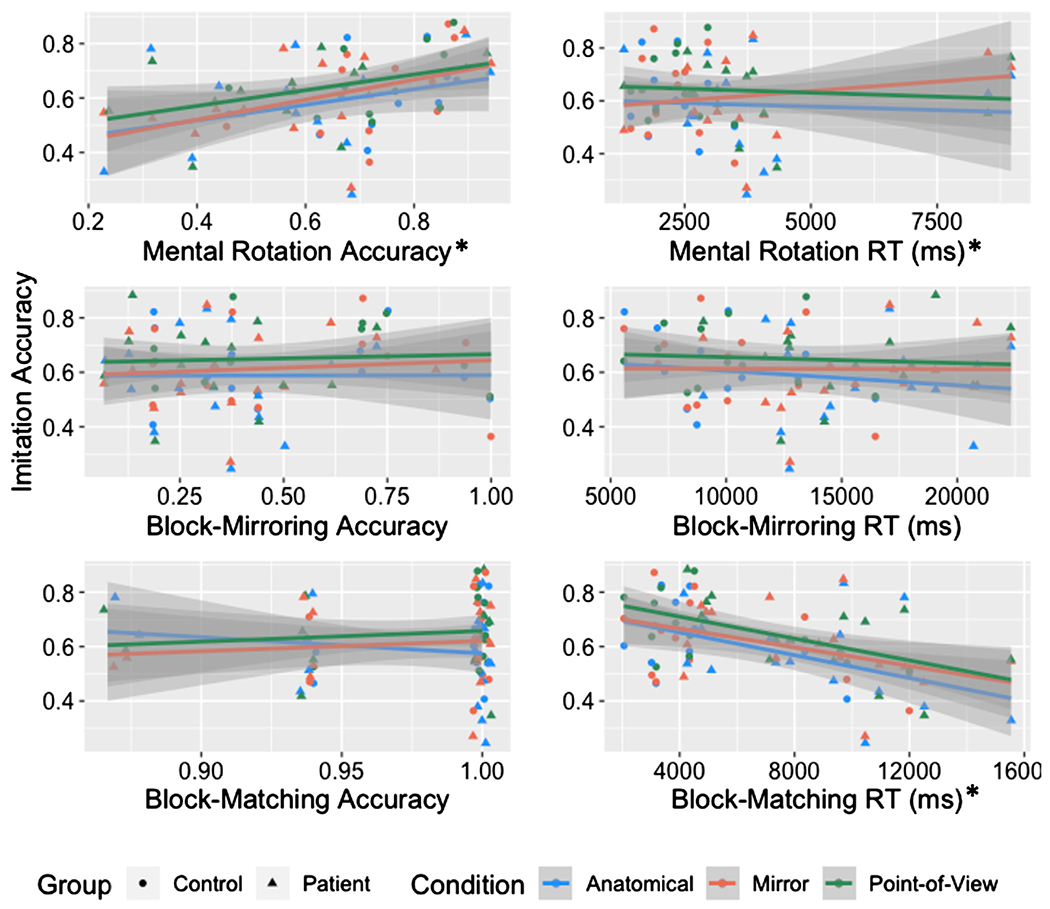
Scatterplot of average imitation accuracy and each visuospatial task (accuracy and RT) with separate lines for condition and separate colors for group. Each individual point represents the average performance of an individual in that condition. Asterisks indicate measures that were significant predictors of imitation accuracy.

For mental rotation accuracy, the model revealed significant effects of mental rotation task accuracy [*χ^2^*(1) = 6.23, *p* = 0.013] and condition [*χ^2^*(2) = 6.32, *p* = 0.043] on imitation accuracy, but no significant interaction of the two [*χ^2^*(2) = 0.64, *p* = 0.727]. As mental rotation accuracy increased, imitation accuracy increased (*B* = 0.3). See Figure 4. For the mental rotation RT analysis, we first removed trials that were incorrect (586 trials), had RTs less than 500 ms (18 trials), or that fell more than 3SD above or below the participant mean (9 trials). There were significant effects of mental rotation RT [*χ^2^*(1) = 6.23, *p* = 0.013] and condition [*χ^2^*(2) = 12.49, *p* = 0.002 on imitation accuracy, but no significant interaction [*χ^2^*(2) = 3.63, *p* = 0.163]^2^.

#### 4.4.2 Block-mirroring

For block-mirroring accuracy, we observed a significant effect of condition [*χ^2^*(2) = 8.96, *p* = 0.011], but no significant effects of block-mirroring accuracy [*χ^2^*(1) = 0.10, *p* = 0.748] or their interaction [*χ^2^*(2) =0.51, *p* = 0.777] on imitation accuracy. For the RT analysis, we first removed trials that timed out (23 cases), the response was quicker than 500 ms (1 case), the response was incorrect (253 cases), or the RT fell more than 3SD above or below the participant’s mean (0 cases). This left a total of 187 trials. There was a significant effect of condition [*χ^2^*(2) = 8.95, *p* = 0.011], but no significant effects of block-mirroring RT [*χ^2^*(1) = 0.27, *p* = 0.606] or the interaction [*χ^2^*(2) = 1.32, *p* = 0.517] on imitation accuracy.

#### 4.4.3 Block-matching

For block-matching accuracy, we observed a significant effect of condition [*χ^2^*(2) = 8.95, *p* = 0.011], but no significant effects of block-matching accuracy [*χ^2^*(1) = 0.01, *p* = 0.916] or the block-matching accuracy*condition interaction [*χ^2^*(2) = 4.81, *p* = 0.090] on imitation accuracy. To assess RT effects, we first removed trials that timed out (2 cases), were incorrect (10 cases), fell 3 standard deviations above or below the mean for each participant (1 case), or were less than 500 ms (0 cases). We then calculated the average RT for each individual. There was a significant effect of block-matching RT [*χ^2^*(1) = 12.07, *p* < 0.001] and condition [*χ^2^*(2) = 8.93, *p* = 0.011] on imitation accuracy, but there was no significant interaction [*χ^2^*(2) = 0.48, *p* = 0.787]. The slower the RTs for block-matching, the lower the imitation accuracy (see Figure 4).

## 5 Discussion

Individuals with LCVA and control participants completed a VR imitation task with three different spatial perspectives: first-person, mirroring, and anatomical. They also performed a limb apraxia task, answered questions about avatar embodiment, and performed three computerized visuospatial tasks meant to share some of the processing requirements of each imitation condition. Using the results of these tasks, we examined three main hypotheses. Our first hypothesis was that accuracy would decrease with increasing spatial transformation requirements across the three conditions. We observed that imitation accuracy was indeed better after observing movement from a first-person point of view than in the anatomical condition (which required a 180° rotation of perspective), though accuracy did not significantly differ between first-person and mirroring or between mirroring and anatomical. Our second major hypothesis was that controls would outperform individuals with LCVA on imitation, and imitation accuracy would be related to limb apraxia in the patient group. Surprisingly, we observed no difference in performance between individuals with LCVA and controls, and no significant relationship with our limb apraxia measure. Finally, our third major hypothesis predicted that imitation in each condition would be related to the relevant background visuospatial task. We primarily observed that imitation accuracy correlated to greater accuracy and faster response times on a mental rotation task, but surprisingly this relationship did not depend on cuing perspective. Together these results replicate the advantage of a first-person perspective in imitation and speak to some of the potential visuospatial processes involved in imitation, but also highlight the limitations of using VR to measure imitation deficits related to limb apraxia. We discuss each of these three hypotheses in greater detail below.

Our findings most strongly support our first hypothesis, that spatial cuing perspective affects imitation ability, though the effect was small and should be interpreted with caution. These results are consistent with prior studies demonstrating advantages for a first-person perspective (Jackson et al., 2006; Krause and Kobow, 2013; Watanabe et al., 2013). The first-person perspective is spatially aligned, with minimal mental transformation requirements. Moreover, especially for our VR paradigm, the first-person perspective is unlikely to induce any sensorimotor interference since the observer and instructor’s movements (assuming imitation is accurate) will look identical to the observer (Brockmole and Wang, 2003; Kessler and Thomson, 2010). Our findings expand on prior work in two key ways. First, instead of viewing the instructor from behind, beside, or viewing only a small portion of the instructor’s body, our VR paradigm allowed the participants to directly observe the movements from a true first-person perspective, with their own avatar appearing to be demonstrating the movements. Previous research indicates that individuals experience body ownership of avatars in virtual environments, especially when the avatar is presented from a first-person perspective (Slater et al., 2008; Slater et al., 2009; Petkova et al., 2011). Results from our embodiment questionnaire were somewhat in line with this. Participants ranged in how strongly they embodied the avatar across conditions, with most reporting at least a moderate level of embodiment. Their qualitative comments indicated that several patients also felt greater embodiment in the first-person perspective. Second, use of VR allowed us to present larger-scale movements that were experienced in a 3-dimensional space, as opposed to viewing 2D videos or being restricted to the use of simple finger-tapping movements. Thus we were able to provide the most direct possible mapping between observed and imitated movements, and consequently we observed that it showed advantages for both individuals with LCVA (including individuals with imitation deficits associated with apraxia) and controls over the third-person anatomical perspective.

Interestingly, we did not observe the predicted advantage for third-person mirroring over anatomical imitation, which contrasted with prior research (Chiavarino et al., 2007; Nishizawa et al., 2015). We hypothesized that the less extreme spatial transformation required in the mirroring condition (a spatial reflection) would result in greater accuracy than the anatomical condition, which required a more effortful 180° rotation (or equivalently, a pair of spatial reflections). Instead, performance in the mirroring condition was not significantly different from either the first-person or the anatomical perspectives. This lack of difference could be explained by the difficulty of performing the task in VR. Accuracy was overall quite low across conditions in both groups, and qualitative results from the embodiment questionnaire suggested that the task felt difficult for the participants in all three perspectives. Thus while the use of VR provided significant advantages in terms of experimental control and manipulation and afforded the ability to test a true first-person perspective, assessing the effects of imitation perspective in a simpler or more familiar environment may have its own advantages, especially for more fine-grained distinctions between third-person perspectives.

Our findings did not support our second hypothesis that we would observe a difference between imitation accuracy in individuals with LCVA compared to controls. This was surprising, as was the lack of a relationship between imitation accuracy and our more conventional measure of limb apraxia. While it is possible that a larger sample size may have made it more likely to detect an effect, we suspect that the lack of a group difference could be related again to the high difficulty of the task—both individuals with LCVA and controls performed fairly poorly with accuracy around 65%. Adding to this difficulty, the VR imitation task also differed from our measure of limb apraxia in that it included a memory requirement (though brief), which was necessary due to the logistics of the first-person condition where participants were viewing their own avatar demonstrate the movement. In the VR task, participants first viewed the instructor’s movement, then performed the movement themselves; this required participants to hold the movement in working memory for several seconds prior to imitating. Our measure of limb apraxia, in contrast, asked participants to watch a video of a movement, then imitate along with a second viewing of that same video. Repetition of the video eliminated the working memory requirement, and thus may be a more sensitive measure of imitation deficits despite the requirement to imitate from a third-person mirroring perspective. Indeed, this measure of limb apraxia has been included in many studies, produces a robust range of scores in individuals with LCVA, correlates with other measures of apraxia, distinguishes individuals with LCVA from controls, and has neuroanatomic data to support its localization to fronto-parietal cortex (Buxbaum et al., 2005; Jax et al., 2006; Buxbaum et al., 2007; Kalénine et al., 2013; Buxbaum et al., 2014; Garcea et al., 2020). Thus, the VR task may not be sufficiently sensitive to limb apraxia. What remains unclear in the current study is whether the difficulty in our VR task arose from lack of familiarity with the VR environment, additional working memory requirements, or some combination of the two. Future research is needed to delineate the interplay of imitation and working memory demands in limb apraxia, and whether VR is a feasible method for doing so. For example, allowing the participant to imitate the movements synchronously along with the virtual instructor is an interesting future direction, but raises questions about how best to do so in the first-person perspective without simply turning the situation into a tracking task. One solution may be a spatial offset, in which the participant’s avatar appears behind the virtual instructor, and the instructor is semi-transparent to enable the instructor limb to be viewed through the instructor body.

Finally, based on our third hypothesis we predicted condition-specific relationships between imitation accuracy and various background visuospatial tasks. Imitation was significantly related to 1) accuracy of mental rotation and 2) reaction time on our novel block-matching task, but there was no relationship between block-mirroring and imitation in any of the conditions. These relationships were not specific to the predicted imitation conditions (anatomical and first-person, respectively), but were instead general predictors across conditions. There may be two potential reasons for this. First, imitation in general may be reliant on visuospatial transformation abilities. Indeed, the relationship between imitation and mental rotation is consistent with theories suggesting that individuals perform visuomotor transformations in imitation tasks, regardless of perspective (Kessler and Thomson, 2010; Creem-Regehr et al., 2013; Watanabe and Higuchi, 2016). Even if this is true, though, it is unclear why we did not see increasing reliance on mental rotation as the disparity between the spatial perspectives of instructor and observer increases. A second possibility is that the relationship between imitation and mental rotation reflects more general cognitive capacities that underlie processing speed and accuracy regardless of task. Supporting this possibility is the observation that the ability to respond quickly on a simple block-matching task also seems to relate to imitation accuracy in a condition-independent manner. Against this hypothesis, however, are the highly accurate performance of all individuals on the block-matching task and the absence of differences between individuals with LCVA and controls on the imitation tasks. Thus many open questions remain regarding the visuospatial factors we assessed and their relationship to imitation, including whether they are specific to predicting imitation in VR versus imitation more generally.

## 6 Limitations

Taken together, we note that many of the findings we observed in this study could be explained by a generic effect of using virtual reality, rather than the experimental manipulation we intended to test. Part of this may be attributed to the fact that almost none of our participants had experienced a VR environment before; individuals with more VR experience may have been more likely to show effects that reflected our standard measure of limb apraxia. Though we incorporated significant practice time prior to the experimental trials, it is also possible that a longer accommodation time in the headset may have strengthened the effect. Moreover, our use of a live tracking system to provide visual feedback of arm movement to the participant in the headset was effective but imperfect. For example, it was sometimes difficult to get the participant to look down enough to observe the instructor’s movement in the first-person condition, and when performing the movements participants sometimes felt like their arm and hand did not look right. In addition, to ensure that participants were able to view the movements in their entirety (i.e., turning their head to watch the movements), movements were quite slow, and many participants commented on how the slowness of the cued movements also made the task feel artificial and the movements hard to imitate. Participants also reported only moderate embodiment of their avatar across all conditions, although qualitatively some participants reported less ability to relate to the instructor avatar in the third-person perspectives than the first-person perspective. This qualitative difference in avatar embodiment could explain in part why we observed the highest accuracy in the first-person perspective, but this idea still needs to be examined more carefully in future research, especially given the linguistic challenges of conducting qualitative interviews with some patients. A more thorough embodiment questionnaire may show greater sensitivity than the brief scale used here, provided it has been validated in individuals with stroke. Finally, it is also possible that a more heterogenous group of patients or a larger sample may have been more likely to show effects, as the effects observed here tended to be small in the current sample.

## 7 Conclusion

In sum, while immersive virtual reality provides advantages for studying imitation behavior in terms of avatar embodiment, experimental manipulation, and control of the first-person perspective, it also has limitations for detecting apraxic behaviors in individuals with LCVA. Though we did not observe a link between imitation in VR and standard apraxia measures, future research with larger samples should address the possibility that practicing imitation in VR could have benefits for rehabilitation more broadly.

## Figures and Tables

**TABLE 1:** Embodiment questionnaire results.

		Individuals with LCVA	Controls
		*M(SD)*	*M(SD)*
Q1. Do you feel like the body you see when you look down is your body?	First-person	1.48(0.85)	1.42(0.90)
	Anatomical	1.29(0.91)	1.36(0.92)
	Mirroring	1.35(0.89)	1.33(0.89)

Q2. When you were performing the movement, did you feel like you could control the virtual body as if it were your own body?	First-person	1.52(0.73)	1.67(0.65)
	Anatomical	1.33(0.92)	1.64(0.81)
	Mirroring	1.43(0.84)	1.67(0.78)

Q3. When you were watching the movement, did you feel as if the virtual instructor was another person?	First-person	1.04(1.02)	1.17(1.03)
	Anatomical	1.46(0.88)	1.45(0.93)
	Mirroring	1.52(0.85)	1.33(0.99)

Note. Each question had a maximum rating of 2. Response options were 0 (No), 1 (Sort of), and 2 (Yes).

**FIGURE A1:**
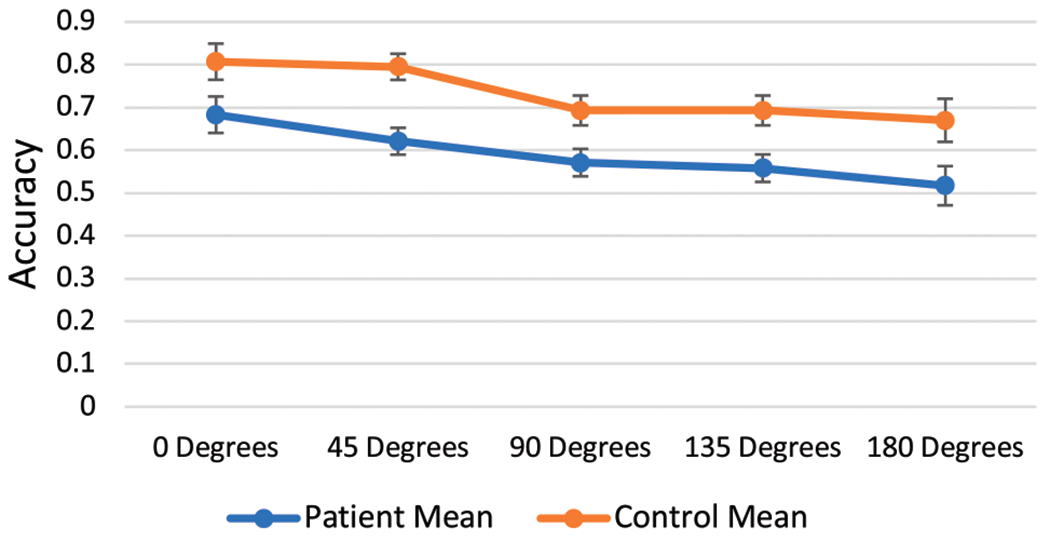
Mental rotation angle of rotation by accuracy.

**FIGURE A2:**
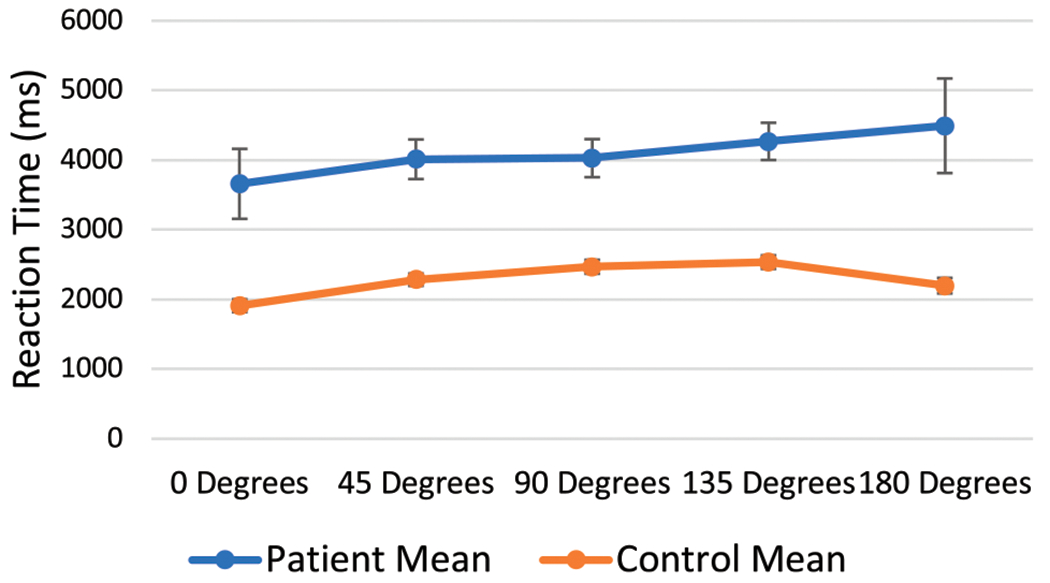
Mental rotation angle of rotation by reaction time.

## Data Availability

The original contributions presented in the study are included in the article/Supplementary Materials, further inquiries can be directed to the corresponding author.

## Footnotes

1 To further explore these effects, we also calculated the average difference between each condition for each participant and calculated Bayes Factors on the *t*-test for each of those difference scores. The alternative hypothesis would be that the difference between conditions is greater than zero. The results of this analysis are consistent with our reported results. The BF for the first-person: anatomical difference is 23.31, or Strong evidence in favor of the alternative hypothesis. The BF for the anatomical:mirroring difference is 0.65 and the BF for the first-person:mirroring difference is 0.30, both considered anecdotal evidence in favor of the null hypothesis.

2 In the mental rotation RT graph, cases on the far right of the graph reflect the average score in each condition of two individuals with LCVA who were particularly slow. We removed those individuals and re-ran the analysis, which reduced the RT effect on imitation accuracy [*χ^2^* (1) = 2.82, *p* = 0.093]. The condition effect remained [*χ^2^* (2) = 7.26, *p* = 0.027], and there was still no significant interaction [*χ^2^* (2) = 3.10, *p* = 0.212]. This suggests that the MRT RT effect was driven mainly by two individuals.

## Appendix

### 7.1 Patient and control differences in visuospatial tasks

Although we did not observe any main effects of group in our imitation task, we nevertheless wanted to examine if there were any group differences in performance on the visuospatial measures. We ran a series of either logistic (accuracy) or log-transformed linear (RT) mixed effects regressions for each measure to test the prediction that controls would outperform individuals with LCVA on all measures. See Table A1 for average performance for each group in each condition.

### 7.2 Mental Rotation

We followed the standard approach in mental rotation analyses to look at angle of rotation as well as our other effects of interest (group). If participants were using a mental rotation strategy (imagining one object rotating to match the other), then accuracy should decrease and RT should slow as angle increases (i.e., the lowest accuracy and slowest RT should be observed at the highest angle). A mixed effects logistic regression model with Angle of Rotation, Group, and Angle*Group predicting accuracy (0 or 1) revealed a significant effect of Angle [*χ^2^*(1) = 18.97, *p* < 0.001] and Group [*χ^2^*(1) = 3.79, *p* = 0.052], but no Angle*Group interaction [*χ^2^*(1) = 0.16, *p* = 0.691]. We included participant as a random effect. As such, accuracy for all participants decreased with increasing angle of rotation, and individuals with LCVA were less accurate than controls. See Figure A1 and Table A1. We then ran the same model for log-transformed RT. We observed significant effects of Angle [*χ^2^*(1) = 22.04, *p* < 0.001] and Group [*χ^2^*(1) = 7.26, *p* = 0.007], but no Group*Angle interaction [*χ^2^*(1) = 0.02, *p* = 0.884]. In sum, as angle increased, reaction time also increased, and individuals with LCVA were slower to respond than controls. See Figure A2 and Table A1. Together, these results suggest that controls outperformed individuals with CVA on mental rotation and participants in both groups demonstrated a similar mental rotation strategy.

### 7.3 Block-mirroring and Block-matching

We first removed trials where the participants did not respond in time (23 trials in block-mirroring, 2 in block-matching). We performed a logistic regression with group predicting accuracy in the block-mirroring task and found a significant effect (*B* = −0.63, *p* < 0.001). Controls were 0.63 times more likely to select the accurate response. The same logistic regression for the block-matching task was not significant (*p* = 0.181). Individuals with LCVA and controls were equally likely to select the correct response, and accuracy was close to ceiling for both groups. We then performed a linear regression with log-transformed RT as the dependent variable and group as the predictor. For the block-mirroring task, group was significant (*B* = 0.40, *p* < 0.001), with individuals with LCVA responding slower than controls. For the block-matching task, group was also significant (*B* = 0.56, *p* < 0.001), with individuals with LCVA again responding slower than controls. Together, these results demonstrate that though both groups performed at ceiling on block-matching, individuals with LCVA were slower and less accurate on all other background tasks, suggesting impaired visuospatial abilities in individuals with LCVA.

**TABLE A1:** Average performance on visuospatial tasks separated by group.

		Individuals with LCVA *M (SD)*	Controls *M (SD)*
Mental Rotation	*Accuracy*	0.59 (0.49)	0.73 (0.44)
	*Reaction Time (ms)*	4,078 (3,664)	2,327 (1,022)

Block Matching	*Accuracy*	0.97 (0.17)	0.99 (0.10)
	*Reaction Time (ms)*	8,442 (4,118)	5,171 (3,852)

Block Mirroring	*Accuracy*	0.36 (0.48)	0.51 (0.50)
	*Reaction Time (ms)*	15966 (6,158)	10736 (5,140)
